# An evaluation of the nature and level of musculoskeletal imaging training in physiotherapy educational programmes in Nigeria

**DOI:** 10.1186/s12909-020-02183-5

**Published:** 2020-08-05

**Authors:** Ogochukwu Kelechi Onyeso, Joseph O. Umunnah, Charles Ikechukwu Ezema, Joseph A. Balogun, Chigozie I. Uchenwoke, Maduabuchukwu Joseph Nwankwo, Kayode Israel Oke, Bashir Bello, Ifeoma Blessing Nwosu, Mishael E. Adje

**Affiliations:** 1 Department of Medical Rehabilitation, Faculty of Health Sciences and Technology, College of Medicine, University of Nigeria, Nsukka, Enugu Nigeria; 2 Department of Medical Rehabilitation, Faculty of Health Sciences and Technology, College of Health Sciences, Nnamdi Azikiwe University, Awka, Anambra Nigeria; 3 Department of Health Studies, College of Health Sciences, Chicago State University, Chicago, USA; 4 Department of Physiotherapy, School of Basic Medical Sciences, College of Medical Sciences, University of Benin, Benin-City, Edo Nigeria; 5 Department of Physiotherapy, Faculty of Allied Health Sciences, College of Health Sciences, Bayero University Kano, Kano, Nigeria; 6 Department of Therapeutic Sciences, Trier University of Applied Sciences, Trier, Germany

**Keywords:** Clinical decision making, Curriculum, Diagnostics imaging, Doctor of physical therapy, Education, Musculoskeletal system, Physiotherapy

## Abstract

**Background:**

Deficiency in musculoskeletal imaging (MI) education will pose a great challenge to physiotherapists in clinical decision making in this era of first-contact physiotherapy practices in many developed and developing countries. This study evaluated the nature and the level of MI training received by physiotherapists who graduate from Nigerian universities.

**Methods:**

An online version of the previously validated Physiotherapist Musculoskeletal Imaging Profiling Questionnaire (PMIPQ) was administered to all eligible physiotherapists identified through the database of the Medical Rehabilitation Therapist Board of Nigeria. Data were obtained on demographics, nature, and level of training on MI procedures using the PMIPQ. Logistic regression, Friedman’s analysis of variance (ANOVA) and Kruskal-Wallis tests were used for the statistical analysis of collected data.

**Results:**

The results (*n* = 400) showed that only 10.0% of the respondents had a stand-alone entry-level course in MI, 92.8% did not have any MI placement during their clinical internship, and 67.3% had never attended a MI workshop. There was a significant difference in the level of training received across MI procedures [χ^2^ (15) = 1285.899; *p* = 0.001]. However, there was no significant difference in the level of MI training across institutions of entry-level programme (*p* = 0.36). The study participants with transitional Doctor of Physiotherapy education were better trained in MI than their counterparts with a bachelor’s degree only (*p* = 0.047).

**Conclusions:**

Most physiotherapy programmes in Nigeria did not include a specific MI module; imaging instructions were mainly provided through clinical science courses. The overall self-reported level of MI training among the respondents was deficient. It is recommended that stand-alone MI education should be introduced in the early part of the entry-level physiotherapy curriculum.

## Background

Physiotherapy profession focuses on maximising movement and functional ability in people having risks of physical impairment and movement dysfunction [1]. To achieve this core objective, physiotherapists need to develop musculoskeletal imaging (MI) competencies for comprehensive assessment of the musculoskeletal system and optimal clinical decision-making [2, 3]. There is evidence that a well-trained physiotherapist can utilise MI appropriately as a direct-access provider or within a multidisciplinary team [4, 5]. Physiotherapists are currently utilising MI in Australia, Canada, the United Kingdom (UK), the Netherlands, Norway, South Africa, and some parts of the United States of America (USA) [2, 5]. In Nigeria, physiotherapists working in a multidisciplinary setting have unrestricted access to patient’s MI films and radiologists’ reports in the case folders [2], but they rely on physicians for MI referral. However, direct-access physiotherapists such as home care providers need to have the competence to refer patients for MI, when necessary [6].

Imaging is of immense importance to physiotherapists in clinical practice, research, and training [3]. For instance, ultrasound scanning (USS) has moved from the radiology suite to point-of-care, where physiotherapists can perform real-time diagnostic musculoskeletal sonography [7, 8]. Magnetic resonance imaging (MRI) scans are useful when examining subtle musculoskeletal pathologies such as stress fractures, which may have consequences for a physiotherapy plan of care [9]. Also, information from dual-energy X-ray absorptiometry (DEXA), scintigraphy and computed tomography (CT) scans can be used for risk assessment to rule out red flags that may contraindicate the use of some physiotherapy modalities [9, 10].

The emergence of direct-access physiotherapy services in many countries [11, 12] and the concomitant progress in physiotherapists’ diagnostic privileges [5] are important factors that have changed the perception about the relevance of MI in physiotherapy education [3, 5, 12]. As a result, some countries have made adjustments in their entry-level physiotherapy curriculum to align with the prevailing standards in physiotherapy education and practices [2, 13, 14]. The physiotherapy programme accreditation standards in the USA and Nigeria now include specific criteria related to diagnostic imaging [2, 15]. Therefore, training in MI procedures such as radiography (X-ray), CT scan, MRI, scintigraphy, USS, and DEXA has become relevant for entry-level physiotherapy programmes to prepare students for direct-access practice [9, 15].

The Nigerian entry-level physiotherapy education is a five-year Bachelor of Physiotherapy or Bachelor of Medical Rehabilitation (henceforth, referred to as BPT programme) [16]. The BPT programme adopts a classical medical model that involves premedical (natural sciences), medical, and core physiotherapy (preclinical and clinical) courses. The BPT curriculum does not have a stand-alone diagnostic imaging course; rather MI instructions are delivered within related clinical courses such as anatomy and orthopaedics [16]. Such clinical courses are termed clinical track courses [13], during which MI instructions are often used to illustrate pathologies and clinical assessment processes [13, 17]. However, a few institutions in Nigeria are introducing stand-alone MI courses that are specifically dedicated to knowledge and skill development about the utilisation of imaging for clinical assessment.

Recently, the National Universities Commission (NUC) approved the transitioning of the BPT programmes to a six-year Doctor of Physical Therapy (DPT) programme as the minimum benchmark for entry-level physiotherapy education in Nigeria [2]. A two-year transitional DPT (t-DPT) programme was also approved to upgrade practitioners with a bachelor’s degree to DPT status. The t-DPT and DPT programmes were designed to deliver MI instructions through stand-alone courses [13]. Physiotherapists in Nigeria who have completed a t-DPT programme abroad will act as faculty members for the t-DPT and DPT programmes scheduled to commence in September, 2020.

Since 1994, all entry-level physiotherapy graduates in Nigeria undergo a compulsory one-year clinical internship in an accredited teaching hospital to obtain a professional licence [18]. The clinical internship involves clinical placements of an intern across required specialities such as MI, orthopaedics, neurology, and general physiotherapy. In contrast to a clinical internship, clinical placement is a (one to four weeks) short clinical rotation or posting for students during BPT training, clinical internship, and postgraduate programmes [19]. Physiotherapists in Nigeria also have the opportunity of acquiring further MI training through continuous professional development (CPD) programmes [6].

The study described the nature of MI instruction in Nigerian physiotherapy educational programmes (entry-level programme, clinical internship, CPD and postgraduate programme) and the self-reported level of MI training received by physiotherapists in Nigeria. Specifically, the following questions were answered: (a) what were the types of MI courses, instructional methods, and qualifications of instructors at each phase of training? (b) When was the first-time MI instruction, and was there any clinical placement and hands-on instruction received by the respondents at each phase of training? and (c) What is the self-reported level of MI training among the respondents? The authors hypothesised that there will be: (a) no significant difference in the levels of training across MI procedures, and (b) no significant difference in the levels of MI training across specialties, years-in-service, levels of qualification, practice settings, and the universities of entry-level programme. The authors anticipated that the study will identify areas of deficiencies in MI instructional approach and help to reconsider its curriculum implications.

## Methods

### Participants and study design

The study was a cross-sectional online survey. The study participants were physiotherapists registered under the Medical Rehabilitation Therapist Board of Nigeria (MRTBN) – a federal agency that regulates physiotherapy education and practice in Nigeria [6]. The agency keeps an up-to-date database of all registered physiotherapists in Nigeria. The authors obtained the permission of the MRTBN to search the database and select potential participants who (a) possessed an entry-level physiotherapy qualification from an accredited university in Nigeria, (b) had completed one-year post-internship clinical experience, and (c) were licensed and practising physiotherapy in Nigeria. Potential participants who had academic or clinical training outside Nigeria or outside the field of physiotherapy were excluded. However, participants with t-DPT qualification were included to allow for comparison between the two forms of entry-level qualifications (BPT and t-DPT). A total of 2308 physiotherapists met the inclusion criteria and were eligible for participation in the study [20]. From this, a sample size of 330 participants was calculated using 95% confidence level and a 5% margin of error [21].

### Research instrument

A validate and reliable Physiotherapist Musculoskeletal Imaging Profiling Questionnaire (PMIPQ) was used for data collection [2]. The PMIPQ has six domains: (a) demographic details, (b) nature of training in MI, (c) level of training in MI, (d) attitude towards MI, (e) utilisation of MI and (f) competence in the use of MI. The PMIPQ was developed and pilot-tested for psychometric properties in a similar population. The test-retest reliability (*rho*-value) across the domains was as follows: C (0.970), D (0.979), E (0.842), and F (0.716) [2].

This study utilised parts A to C of the PMIPQ. Part A obtained demographic information such as age, sex, years-in-practice, practice setting, speciality, educational background, and CPD. Part B collected data on the characteristics of the respondent’s MI training during entry-level programme, internship, workshop, and postgraduate programme (the type of courses, first-time instruction, teaching methods, qualification of MI instructor, clinical placement, and hands-on experiences). Part C collated the respondent’s level of training on referral and utilisation of MI results. The term “level of MI training” represents the self-reported quality of MI education received by a respondent. The respondents rated the questions in Part C using a 5-point Likert scale ranging from 5 (excellent) to 1 (poor). The full-version PMIPQ can be accessed on 10.4102/sajp.v75i1.1338.

### Procedures for data collection

The authors transcribed parts A to C of the PMIPQ to an online survey. The 2308 eligible physiotherapists were sent an email invitation to participate in the survey. The email contained the objectives of the study and the survey link. On accessing the survey link, respondents first went through an informed consent page before proceeding to the main questionnaire. The consent page included information about the survey and a closing-statement asking the participants if they were willing to participate in the study. A participant had to click on the “yes” button before proceeding to the survey or choose the “no” button to abandon the survey. Therefore, the return of the completed survey constituted consent to participate in the study. Non-respondents received three successive reminders: after two days, four days, and one week of the initial request.

To minimise the incidence of missing data, the questionnaire was programmed to redirect the participants to complete all the compulsory fields before the survey could be submitted. A successful submission was followed by a congratulatory message. Similarly, programming syntaxes were embedded in the software to analyse the demographic variables and discard entries from ineligible respondents or multiple entries from eligible respondents. The instrument was hosted online for 30 days, between March and April 2019.

### Data analysis

The data were analysed with Statistical Package for the Social Sciences (SPSS, Chicago, IL, USA) Version 22 software. Descriptive statistics including frequency, percentage, median, mean and standard deviation were used to describe the nature of MI instructions received by the respondents.

Respondents’ total score on the six MI procedures was computed as their overall level of MI training (range, 6 to 30). The data were screened for normality using the Shapiro-Wilk’s test and Mauchly’s test to determine whether the assumption of sphericity was met. The findings revealed that the data violated the assumptions of normality and sphericity. Consequently, non-parametric inferential statistical methods were applied.

Friedman’s *analysis of variance (*ANOVA) and a pair-wise Wilcoxon signed-rank (Bonferroni adjusted) post hoc test were used to determine any significant difference in the level of training across the MI procedures. Similarly, Kruskal-Wallis test was used to examine if there was a difference in the overall level of MI training across subcategories of the demographic variables. Dunn-Bonferroni post hoc test was applied when a significant difference was obtained. Finally, logistic regression was employed to analyse the interactions between the demographic variables and the overall level of MI training. The level of significance was set at an alpha level of 0.05.

### Ethical consideration

Before the commencement of the study, the authors obtained ethical approval from the Health Research and Ethics Committee of the Faculty of Health Sciences and Technology, Nnamdi Azikiwe University, Nnewi Campus, Nigeria (ERC/FHST/NAU/2018/198). The participants granted their individual informed consent before proceeding to the survey.

## Results

Of the 2308 emails sent out to the eligible candidates, 2125 successfully delivered. Four hundred and thirty-six responses were received, accounting for 20.5% response rate. Only 400 responses were deemed to be complete and were included in the analysis. Most of the respondents were male (*n* = 275, 69.0%) than females (*n* = 125, 31.0%). The mean age of the respondents was 33 ± 8 years, and the mean practice experience was 8 ± 7 years. Other demographic variables are shown in Table 1.

**Table 1:** Level of MI training across the participant’s demographic profile

Parameter	ƒ (%)	Mean Rank	Kruskal-Wallis (***H***)	***P***-value
**Speciality of Interest**				
Orthopaedics/Musculoskeletal	185 (46.3)	201.30	19,737.00^b^	0.897
Non- Orthopaedics	215 (53.7)	199.81		
**Years in Practice**				
1–10	324 (81.0)	192.88	8.50	0.014*
11–20	49 (12.3)	223.14		
21–30	27 (6.7)	250.81		
**Highest Qualification**				
Bachelor of physiotherapy ^a^	280 (70.0)	194.64	12.867	0.005*
Transitional DPT ^a^	8 (2.0)	304.31		
Master of physiotherapy	89 (22.3)	195.20		
Doctor of Philosophy (Ph.D.)	23 (5.7)	256.30		
**University of BPT Training**				
University A	36 (9.0)	243.96	14.489 ^c^	0.025*
University B	48 (12.0)	221.94	5.501^d^	0.358
University C	168 (42.0)	189.32		
University D	52 (13.0)	189.77		
University E	38 (9.5)	221.91		
University F	35 (8.7)	162.30		
University G	23 (5.8)	216.46		
**Practice Setting**				
Federal Hospital	124 (31.0)	214.45	7.320	0.396
State/General Hospital	74 (18.5)	194.62		
Private Hospital	60 (15.0)	185.08		
Private Physiotherapy Clinic	64 (16.0)	201.14		
Home Physiotherapy Service	33 (8.3)	183.50		
Sports Team	6 (1.5)	163.58		
University	19 (4.7)	240.53		
Others	20 (5.0)	181.08		

The universities were anonymised for ethical reasons. *DPT* Doctor of Physical Therapy, *BPT* Bachelor of Physiotherapy, ^a^ = entry-level programme. ^b^ = Mann-Whitney U Statistics. ^c^ = Unadjusted (*n* = 400). ^d^ = Adjusted for other demographic variables (*n* = 145). Dunn-Bonferroni Post Hoc test was reported in-text. Levels of significance (2-tailed): *p* < 0.05 (*)

Three-quarter of the respondents reported receiving MI instructions during their entry-level training programme (BPT). Specifically, those respondents (74.3%) reported that instruction about MI commenced in the first (2.3%), second (5.7%), third (22.5%), fourth (29.0%), and the final year (14.8%) of the BPT programme. A little over half (*n* = 257) of the respondents reported that their MI training was delivered as clinical track courses during their BPT education. Of the 257 respondents, 97.7% had received 1 to 6 clinical track courses, while 2.3% reported to have received more than 6 clinical track courses. Theory only (28.5%) or both (theory and practical-35.5%) was the highest reported form of MI instructional method. Most of the respondents (80.0%) reported that either a physiotherapist or a radiologist or both taught them MI. Forty-two percent did not have clinical placement in the Diagnostic Imaging Department during their BPT programme (Table 2).

**Table 2:** Characteristics of the MI training received by respondents

Parameter *n* (%)	Undergraduateprogramme 400 (100%)	Internship programme 400 (100%)	Workshops 400 (100%)	Postgraduate programme 112 (100%) ^a^
**Type of courses offered**				
Stand-alone course	40 (10.0)	–	–	0 (0.0)
Clinical track courses	203 (50.8)	206 (51.5) ^b^	–	52 (46.4)
Both	54 (13.5)	–	131 (32.7) ^d^	0 (0.0)
Not taught	103 (25.7)	194 (48.5)	269 (67.3)	60 (53.6)
**Instructional method**				
Theory only	114 (28.5)	48 (12.0)^c^	31 (7.7)	21 (18.7)
Practical only	41 (10.3)	110 (27.5)	30 (7.5)	24 (21.4)
Both	142 (35.5)	48 (12.0)	70 (17.5)	7 (6.3)
Not taught	103 (25.7)	194 (48.5)	269 (67.3)	60 (53.6)
**Teaching personnel**				
Physiotherapist	105 (26.3)	156 (39.0)	12 (3.0)	35 (31.2)
Radiologist	88 (22.0)	6 (1.5)	79 (19.7)	10 (8.9)
Both	88 (22.0)	32 (8.0)	40 (10.0)	7 (6.3)
Others	16 (4.0)	12 (3.0)	0 (0.0)	0 (0.0)
Not taught	103 (25.7)	194 (48.5)	269 (67.3)	60 (53.6)
**Clinical placement in DID**				
Yes	232 (58.0)	29 (7.2)	–	10 (8.9)
No	168 (42.0)	371 (92.8)	–	102 (91.1)
**Hands-on training in USS**				
Yes	18 (4.5)	18 (4.5)	38 (9.5)	5 (4.5)
No	382 (95.5)	382 (95.5)	362 (90.5)	107 (95.5)

Clinical track courses = Clinical sciences such as anatomy and orthopaedics that are not focused on MI, but through which MI could be discussed while illustrating pathologies [13]. *DID* Diagnostic Imaging (clinical) Department, *USS* Ultrasound scan

^a^ = Only 112 of the 400 respondents hold a postgraduate qualification

^b^ = Respondents that were taught MI while on internship under senior colleagues

^c^ = Respondents that were taught MI through internship seminars

^d^ = Respondents that attended at least a workshop with main- or sub-topic on MI

- = Data was not obtained or not applicable

Most of the respondents (68.0%) were trained on how to incorporate MI in clinical decision-making during the mandatory internship programme. Clinical physiotherapists delivered MI instruction during the internship programme, according to 39.0% of the respondents. A total of 92.8% did not undergo MI clinical placement in the Diagnostic Imaging Department during internship. Among the 112 respondents with a postgraduate degree, 53.6% reported that they were not taught or that an imaging course was not a part of their programme. A little over quarter (*n* = 131 of 400) reported to have attended workshops on MI. Of the 131 respondents, 76.3, 15.3, and 8.4% reported to have attended 1, 2, or 3 workshops, respectively. The majority (95.5%) were not taught musculoskeletal USS at bachelor-level, internship or during postgraduate training (Table 2).

The median (range 1 to 5) rating of the levels of training across various MI procedures was as follows: X-ray = 3 (good); MRI and CT scan = 2 (fair); USS, scintigraphy, and DEXA = 1 (poor). Friedman’s ANOVA (Table 3) showed that there was a significant difference in the level of training received across the MI procedures (χ^2^ (15) = 1285.90, *p* < 0.001). Wilcoxon signed-rank Bonferroni-adjusted post hoc analysis showed a significant difference in the level of training between the following MI procedures: *X-ray* versus MRI (*p* < 0.001), CT scan (*p* < 0.001), USS (*p* = 0.015), scintigraphy (*p* < 0.001), and DEXA (*p* < 0.001); *MRI* versus CT scan (*p* < 0.001), USS (*p* < 0.001), scintigraphy (*p* < 0.001), and DEXA (*p* < 0.001); *CT scan* versus USS (*p* < 0.001), scintigraphy (*p* < 0.001), and DEXA (*p* < 0.001). However, there was no significant difference between the level of training received on *USS* versus scintigraphy (*p* = 4.80) or DEXA (*p* = 0.15), and *scintigraphy* versus *DEXA* (*p* = 0.11).

**Table 3:** Friedman ANOVA: differences in the level of training in MI modalities

Diagnostic Imaging Modality.	***N***	Mean Rank	Friedman Test [χ^2^(15)]	***P***-value
Radiography (X-ray)	400	5.63	1285.899	0.001**
Magnetic Resonance Imaging (MRI)	400	4.09		
Computed tomography (CT Scan)	400	3.72		
Ultrasound Scan (USS)	400	2.61		
Scintigraphy (bone scan)	400	2.55		
Dual Energy X-Ray Absorptiometry (DEXA)	400	2.40		

Levels of significance (2-tailed): *p* < 0.05 (*).Wilcoxon signed-rank Bonferroni Adjusted Post Hoc test was reported in-text

Table 1 showed the differences in the overall level of MI training across demographic variables. A Kruskal-Wallis test showed no significant difference across specialities of interest, and practice settings. The dataset was sorted to select respondents (*n* = 145) who had the same demographic variables except the university of entry-level-education. Kruskal-Wallis was run on the subset. The outcome showed that there was no significant difference in the level of training reported across the Universities.

However, there was a significant difference across years-in-practice (*H =* 15.76, *p* = 0.008). The Dunn-Bonferroni pair-wise post hoc test showed a significant difference between respondents who had practised for a decade or less and those who had practised between two to three decades (*Z* = − 128.94, *p* = 0.05); there was no significant difference between the other pairs. Similarly, there was a significant difference between the level of training in MI across various educational qualifications (*H* = 12.867, *p* = 0.005). The post hoc test showed a significant difference between the respondents with t-DPT and BPT qualifications (*Z* = − 109.67, *p* = 0.047).

Furthermore, a binary logistic regression was used to explore the interactions between all demographic variables and the overall level of MI training (as the dependent variable). The outcome showed that the academic qualification only had a statistically significant interaction with the dependent variable. The odds of t-DPT holders having a higher level of MI training was 0.121 (95% CI: 0.038 to 0.391) times that of BPT holders (Wald χ^2^ (1) = 12.48, *p* < 0.001).

## Discussion

The emergence of direct-access physiotherapy practices and the concomitant diagnostics referral rights [5, 12] have changed the perception about the importance of MI in physiotherapy education worldwide [3, 5]. This justifies the exploration of the nature and level of MI education among physiotherapists in Nigeria. The PMIPQ enquired about physiotherapist’s MI education at the bachelor programmes, clinical internship, CPDs, and postgraduate programmes, to discover areas of deficiencies.

The demographic findings were similar to another online survey conducted among the Nigerian physiotherapists. Adje and colleagues [22] reported that 42.5% of physiotherapists in Nigeria were in the musculoskeletal speciality, but this study found a slightly higher percentage, 46.3%. The increase could be attributed to the subject area (MI), which generally influences the response rate in an online survey. The average age of the respondents in the previous study (32 ± 6 years) [22] was similar to that of the present study (33 ± 8 years). This study agrees with Balogun and colleagues [23] who found that two-third of physiotherapists practising in Nigeria were men. The present study reports a wide range of practice years, but the categorisation of the respondents under decades of years-in-practice showed that the majority (81.0%) of the respondents had practised for just one decade or less. Therefore, the outcome of this study fairly represents the present situation of MI education within the study population.

There was a paucity of literature on the essence of physiotherapists’ diagnostic and procedural imaging training. This study draws most of its comparison from a USA-based study by Boissonnault and colleagues [13], which appears to be the only published study on the nature of MI curriculum in a countrywide physiotherapy programme.

In Nigerian BPT curriculum [16], MI instructions were imbedded in multiple clinical science courses. Only a few institutions have proactively included a specific MI course. In contrast, half of the USA-based entry-level physiotherapy institutions have included specific stand-alone MI courses in their curriculum [13]. Apart from the curriculum content, the nature of entry-level MI training can be characterised by first-time instruction [13], the qualification of the MI instructor [17], duration of classroom exposure and clinical placements [19, 24]. Nearly all the USA-based institutions introduced imaging content in the first or second year of their entry-level DPT programme [13]. Conversely, majority of the respondents in the present study received first-time MI instruction during the penultimate year of their BPT programme. The discrepancy can be attributed to the differences between the BPT and DPT curriculums. The American Physical Therapy Association recommended an early integration of MI knowledge and skills in the DPT educational programme in tandem with the clinical track courses [15]. Further studies are necessary to provide empirical evidence on the effects of early and late introduction of MI instructions on the clinical competence of pre-licensure physiotherapists. Meanwhile, as Nigerian institutions prepare to implement the DPT programme with a stand-alone diagnostic imaging module [2], the MI instructions should be introduced early in the entry-level programme.

Classroom activities and hospital-based clinical placement are vital components of the entry-level physiotherapy programmes worldwide [19, 24]. The majority of the respondents reported that they received a clinical placement at the diagnostic imaging department of the affiliated hospitals during their BPT programme. This finding is similar to practices in the USA, where entry-level physiotherapy students receive MI instruction during hospital placement [24]. The qualification of the MI instructors is equally important. The respondents were mostly lectured by physiotherapists, radiologists or both. The authors are not aware of any other studies that have investigated the qualification of the lecturer who teaches MI contents in the entry-level physiotherapy programme. Chojniak and colleagues [17] stated that the qualification and supervisory qualities of clinical trainers are essential characteristics in the effectiveness of diagnostic imaging teaching and learning. Moreover, MI is a technical subject that requires an instructor with related qualification, who has the skills and experience to blend the theory and clinical application while delivering the contents. In this context, a physiotherapist with postgraduate qualification in diagnostic imaging or a radiologist would be the most appropriate MI instructor for physiotherapy students.

Most of the respondents did not have diagnostic imaging placement during their post-BPT clinical internship. It implies that only a few internship programmes (hospitals) have started diagnostic imaging clinical placement for physiotherapy interns. Sak-Ocbina and colleagues [24] recommended that physiotherapy students should be exposed to diagnostic imaging during full-time clinical internships. In view of the emerging direct-access and MI privileges among physiotherapists across the world, internship training should be enhanced where applicable. The physiotherapy training institutions should equip their interns with commensurate MI training for post-internship independent practices.

Similarly, none of the survey respondents had stand-alone MI instruction or clinical placement during postgraduate programmes (*n* = 112). Postgraduate physiotherapy training in Nigeria involves research work, speciality and elective courses [25], that do not include MI instructions. The finding is different from the situation in Canada where postgraduate physiotherapy programmes have integrated MI courses. Chong and colleagues [26] report that some Canadian physiotherapists have taken postgraduate courses related to diagnostic imaging (X-ray, MRI, CT, and USS). Physiotherapists with postgraduate qualification in diagnostic imaging may serve as faculties, delivering MI instruction at entry-level programmes, clinical internships and workshops. Furthermore, postgraduate programmes should develop advanced competencies in rehabilitative MI procedures such as ultrasound-guided interventions, which are beyond the entry-level programme [8].

Surprisingly, most respondents had never participated in any diagnostic imaging workshop. The few respondents who had attended such workshops did not have hand-on MI experience. Although, the MRTBN has made participation in CPD programmes a prerequisite for the annual renewal of physiotherapy practising licence in Nigeria, a question of interest asked was if these CPDs incorporate MI contents. There is a paucity of international literature on MI instructions during physiotherapy CPD programmes. Potter and colleagues [8] reported that musculoskeletal ultrasound workshops are scarce for physiotherapists in the UK. Therefore, the authors wish to draw to the attention of the physiotherapy CPD providers to the need to integrate hand-on MI instructions in continuing education programmes, to enhance participant’s clinical decision-making skills. Specifically, USS workshops should be prioritised. Musculoskeletal sonography is a promising area in international physiotherapy practice due to its availability, safety, sensitivity for muscle and joint pathologies, and cost-effectiveness [7, 8, 27]. As physiotherapy practice scope, and MI competence is expanding, the present authors align with other scholars [2, 8, 15] who have advocated for the upgrade of USS contents in physiotherapist’s training.

Another objective of this study was to explore the level of training across MI procedures. The respondent’s self-reported rating for X-ray was significantly higher than all other MI procedures. The rating for MRI and CT scans were moderate, while USS, scintigraphy, and DEXA were low. The findings concur with a USA-based study that reported that training in X-ray and MRI were higher among students when compared with training in other imaging modalities [24]. Similarly, Boissonnault and colleagues [13], reported that the USA faculties’ perceived their students to be more competent in recognition of pathologies from an X-ray, followed by MRI, CT scan, scintigraphy and USS, respectively. The authors could not find any publications on physiotherapists’ extent of training in DEXA procedure. Nonetheless, physiotherapists use DEXA for research and clinical decision-making [10].

The respondents with t-DPT reported a higher level of MI training than their counterparts with BPT. The t-DPT and DPT programmes were designed to provide adequate imaging education to prepare new graduates for diagnostic imaging privileges [24]. The implementation of the DPT programme in the USA resulted in an increased emphasis on imaging modules [13]. The advocacy for entry-level DPT programmes has been ongoing in Nigeria for over two decades [25]. Fortunately, the NUC approved the DPT curriculum in 2018, and the emphasis has shifted to speedy implementation. A DPT readiness evaluation conducted among all the Nigerian physiotherapy institutions (as at 2017, *n* = 7) showed that the majority of the universities were ready for the curriculum-upgrade [23].

The regression output showed that years in service, university of BPT training, specialty of interest, and practice setting did not have any significant effect on the overall level of MI training. This outcome aligns with other findings: virtually all the respondents have practised for less than two decades. Almost all the universities did not include a stand-alone MI course, they relied on clinical track courses for MI instructions [16]. Although postgraduate academic specialisation is available, specialty-based clinical-residency training has not commenced in Nigeria. Irrespective of postgraduate training, clinicians are allowed to practise under their specialties of interest. Nonetheless, by the nature of their profession, physiotherapists of all specialties are expected to be competent in musculoskeletal assessment. Since direct-access musculoskeletal providers are predominant, the expectation of independent practice competence gives further justification on the need for proper MI training.

Literature has shown the relevance of MI in the emerging direct-access physiotherapy in Nigeria [6], and across the world [4, 5]. Consequently, this study evaluated and revealed areas of deficiencies in the MI instructional approach among Nigerian physiotherapy programmes. The authors have discussed several ways to improve the MI education. In addition, the findings of the study suggest that implementation of the DPT programme may lead to an improvement in the MI curriculum content and instructional approaches.

### Limitations

The study participants were not randomly selected, which could lead to distribution bias, affecting the generalisation of the findings to all physiotherapists in Nigeria. The few t-DPT holders (*n* = 8) who participated in this study had a BPT and clinical internship in Nigeria, however, they obtained t-DPT abroad. They were included to allow the authors to compare the extent of MI training among physiotherapists with only a Bachelor’s degree and those with additional t-DPT.

The study instrument was designed to generate subjective data based on a self-reported recall of the respondents’ training experiences, using a single measure, a five-point Likert scale. Although the instrument captured the aspect of continuous professional development, the authors could not rule out all possible interfering exposures during the timeline. Moreover, some respondents may not remember past events accurately – a common limitation of questionnaire-based studies. Additionally, some parts of the survey were programmed as compulsory fields this reduces a participant’s freedom and may contribute to a low response rate.

On the nature of MI training, the PMIPQ cannot obtain information on the quantity of MI instruction during the entry-level classroom lectures, hospital placements, clinical internship, and postgraduate programme. Further studies should be institution-based and focus on MI contents and curricular performance; faculties may be asked to describe their curricula in detail.

## Conclusion

Majority of the respondents indicated that their entry-level (BPT) education and postgraduate programme did not offer core stand-alone imaging modules. Instead, MI instruction was mainly delivered through multiple, related clinical courses. Most of the MI instruction was provided during the penultimate year of entry-level education. The majority of the respondents did not receive sufficient MI training. The level of training in X-ray was significantly higher than the levels of training in MRI and CT scans, which were significantly higher than the levels of training in USS, DEXA, and scintigraphy, in that sequence. Educational programmes may adopt a specific MI course in the early part of their curriculum, with clinical placements and hands-on instructions. MI instructors should possess basic diagnostic imaging qualification. MI contents should be incorporate in the clinical internship and CPD programmes.

## Data Availability

The questionnaire used and datasets analysed during the current study are available from the corresponding author on reasonable request.

## Abbreviations

ANOVA: Analysis of Variance
CPD: Continuous Professional Development
CT scan: Computed Tomography Scan
DEXA: Dual Energy X-ray Absorptiometry
DPT: Doctor of Physical Therapy Programme
M.Sc.: Masters of Science Degree
MRI: Magnetic Resonance Imaging
MRTBN: Medical Rehabilitation Therapists Board of Nigeria
NUC: National Universities Commission, Nigeria
Ph.D.: Doctor of Philosophy Degree
PMIPQ: Physiotherapist’s Musculoskeletal Imaging Profiling Questionnaire
t-DPT: Transitional Doctor of Physical Therapy Programme
UK: United Kingdom
USA: United States of America
USS: Musculoskeletal Ultrasound Scan
X-ray: Radiography
